# An Integrated Pressure-Volume Loop and Pulmonary Artery Catheter

**DOI:** 10.1016/j.jacbts.2025.101326

**Published:** 2025-07-30

**Authors:** M. Imran Aslam, Aleksandra B. Gruslova, Luis A. Diaz Sanmartin, Drew Nolen, James J. Elliott, Alexander J. Moody, Joel E. Michalek, Vivek P. Jani, Fawaz Alenezi, Clay Heighten, Jonathan W. Valvano, Marc D. Feldman

**Affiliations:** a Division of Cardiology, Department of Medicine, Duke University Medical Center, Durham, North Carolina, USA; b Department of Biomedical Engineering, University of Texas at San Antonio, San Antonio, Texas, USA; c Department of Laboratory Animal Resources, University of Texas Health Science Center at San Antonio, San Antonio, Texas, USA; d Research Imaging Institute, University of Texas Health Science Center at San Antonio, San Antonio, Texas, USA; e Department of Population Health Sciences, University of Texas Health Science Center at San Antonio, San Antonio, Texas, USA; f Division of Cardiology, Department of Medicine, Johns Hopkins University School of Medicine, Baltimore, Maryland, USA; g CardioVol LLC, Dallas, Texas, USA; h Department of Electrical and Computer Engineering, University of Texas at Austin, Austin, Texas, USA

**Keywords:** admittance, pressure-volume loops, pulmonary artery catheter, RV volume

## Abstract

An admittance technology (AT) pulmonary artery catheter was developed to measure instantaneous right ventricular (RV) pressure-volume loops. Admittance RV volumes were validated in vivo through 10 swine subjected to positive and negative hemodynamic perturbations compared with magnetic resonance imaging and ex vivo RV foam casts. Finite element analysis demonstrated the AT electric field was confined to RV blood volume. RV pressure-volume loops demonstrated anticipated changes in all hemodynamics, and end-diastolic volumes were equal to magnetic resonance imaging and foam casts (*P =* NS). Increasing RV end-diastolic volumes in response to acute cardiac injury were also measured (*P <* 0.001). We report validating an AT pulmonary artery catheter in a swine model.

## Highlights

• An admittance technology PAC was developed to measure instantaneous RV pressure-volume relationships.
• Absolute admittance RV volumes were shown to be accurate compared with the standards of CMR and ex vivo RV foam casts.
• RV-PV loops demonstrated the anticipated hemodynamic responses to intravenous dobutamine, intravenous phenylephrine, and intracoronary RCA microspheres.
• We report validating an admittance PAC in swine.

## Visual Abstract

**Figure:**
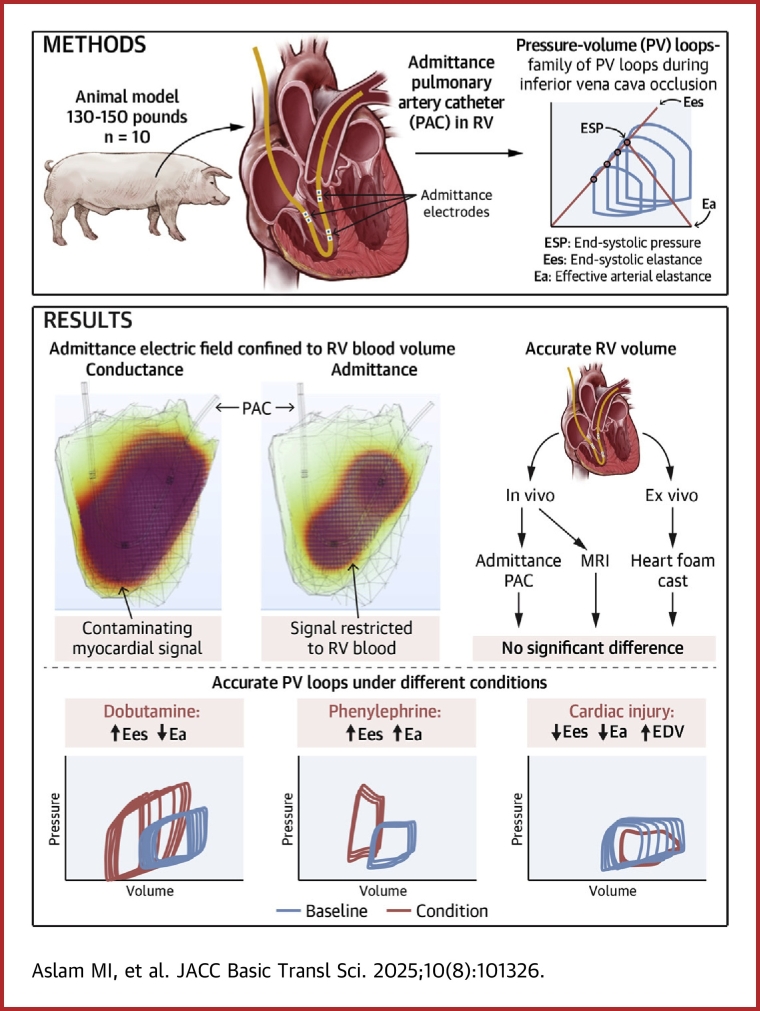

The pulmonary artery catheter (PAC) was originally introduced in 1970 by Swan and Ganz.^1^ It has led to the hemodynamic characterization of several cardiac and pulmonary disease states including, but not limited to cardiogenic shock, pulmonary hypertension, right ventricular (RV) dysfunction, acute respiratory distress syndrome and pericardial diseases.^2^^,^^3^ Despite its importance in clinical medicine, there has been no evolution in catheter design since its original description. Currently, pressures and cardiac output are routinely measured. However, PACs are limited in their inability to measure RV volumes, which provides a critical insight into chamber function. Assessment of chamber volumes, coupled to invasive pressures, facilitates understanding of ventricular compliance, interaction with pulmonary artery (PA) loading and ultimately an understanding of myocardial function not afforded by routinely used methods (ie, PAC measurements and echocardiography).

The addition of RV volume measurement to a PAC would provide the ability to construct pressure volume (PV) loops. PV loops as a measure of cardiac mechanics were developed in the 1970s by Suga et al^4^ to allow determination of cardiac chamber function independent of changing preload and afterload. These measures include end-systolic elastance (Ees) as a measure of systolic function, effective arterial elastance (Ea) as a measure of the load the chamber pumps against, and their ratio (Ees/Ea) to determine how well systolic function and load are coupled. Traditionally, many of the published reports in the PV loop domain on ventricular-vascular coupling, contractility, and afterload have been applied to the left ventricle (LV).^5^, ^6^, ^7^ Bringing the powerful construct of PV loops to the RV would be an important advance, especially if this capability were retrofitted onto an existing PAC, which is used ubiquitously.

The largest hurdle to RV volume measurements is the complexity of the shape of the RV. The RV is shaped like a crescent wrapping around the LV, without discrete geometric dimensions. During pressure and/or volume overload, the RV dilates and remodels by becoming more globular in shape. Although there are several other technologies suited to obtain RV volume, such as 3-dimensional (3D) echocardiography and cardiac magnetic resonance (CMR), neither is obtained with ease, or coupled with simultaneous determination of RV pressure on a routine clinical basis.

The solution proposed in the current study is the use of electrical fields as a method to obtain accurate real-time RV volumes. Electric fields are an ideal solution because they fill any shaped chamber. The traditional method, termed *conductance*, is contaminated by signal from the myocardium, being unable to differentiate between muscle and blood components. We solve this problem by utilizing complex admittance, developed by our group.^8^, ^9^, ^10^ The RV is the ideal application for admittance because it can account for the complexity of its shape, particularly as the RV remodels in various disease states.

## Methods

### The admittance technique

To measure instantaneous RV volumes, the admittance technique was employed. The admittance technique has been extensively studied and previously validated in patients when compared with 3D echocardiography in the RV.^11^ Admittance measurements were performed in tetrapolar configuration by injecting 100 μA RMS at 20 kHz at the distal pair and measuring voltage response from the inner 2 electrodes. The admittance technique accounts for the capacitive properties of muscle, isolating the blood component from the complex bulk measurement algebraically. At 20 kHz, only muscle provides a susceptance measurement, allowing us to measure blood conductance directly from the real and imaginary parts of the admittance measurement.^12^^,^^13^

Working beyond Baan’s equation for translating conductance measurements inside ventricles to volume,^14^ Wei et al^12^ described in larger volumes, that a nonlinear relationship exists between a chamber’s volume and its blood conductance. Wei defines G_inf_ as the conductance measured at infinite volume (S).

(1) $$SV=\left(\rho \times L^{2}\right)\times \left(\frac{G_{\inf}\times Gb_{ED}}{G_{\inf}-{Gb}_{ED}}-\frac{G_{\inf}\times Gb_{ES}}{G_{\inf}-Gb_{ES}}\right)$$

Here, ρ is blood resistivity (Ω∗m), L is the distance between voltage measuring electrodes (m), and Gb is the measured conductance at end diastole (Gb_ED_) and end systole (Gb_ES_). Wei took ρL^2^ as a known constant and used a single stroke volume (SV) calibration to determine G_inf_.

To account for the complex morphology of the RV, we modified the calibration process to use 2 independent measurements. First, we directly measured G_inf_ by placing the catheter in an 18-L saline reservoir. We matched the conductivity of the saline to the conductivity of the blood. Second, we measured SV (by thermodilution). Now, the terms G_inf_ and *ρL*^*2*^ are 2 calibration constants. Wei’s equation becomes:

(2) $$V=\left(\rho \times L^{2}\right)\times \left(\frac{Gb}{1-\frac{Gb}{G_{\inf}}}\right)$$

Having an independent measurement of SV (by thermodilution) and an independent measurement of G_inf_, we can calculate 2 calibration constants, minimizing the uncertainty introduced in Wei’s equation by catheter bending and electrode position in the RV. Given the admittance catheter area of sensitivity focused on the right ventricular outflow tract (RVOT) and apex, we calibrated the SV input of Wei’s equation to represent 60% of the total thermodilution SV.^15^

### Catheter design

**Figure 1:**
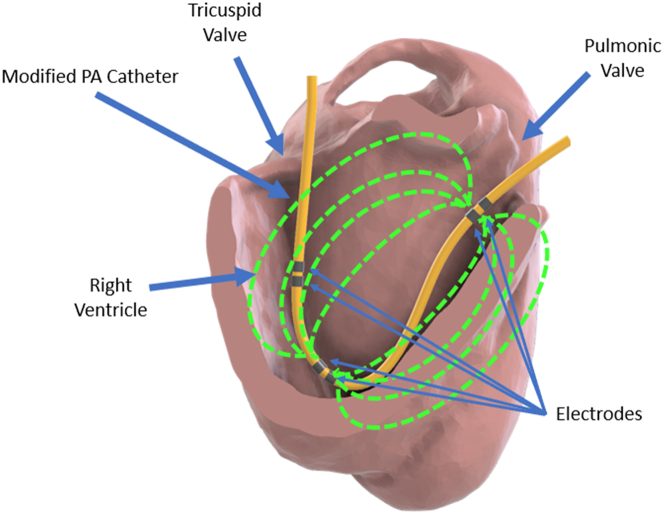
PA Catheter Modified With Admittance Electrodes Inside the Right Ventricle Green dotted lines represent the electrical field created inside the right ventricular chamber. PA = pulmonary artery.

We propose an electrode configuration that optimizes electrical field distribution in the inflow tract or the outflow tract, creating 2 effective measuring fields. Ideally, an admittance RV catheter would include inflow and outflow chambers. However, 2 independent admittance circuits would be required, as well as an understanding of the interaction between the 2 simultaneous intraventricular electric fields. To simplify this problem, the current study focused only on the RVOT electric field utilizing the distal electrode pair sitting below the pulmonic valve and the apex only as shown on Figure 1.

A standard 7-F PAC was modified by Transonic Scisense Inc. For acquisition of data in live animals, a custom admittance control unit (University of Texas), and a BridgeAmp (AD Instruments) were used.

**Figure 2:**
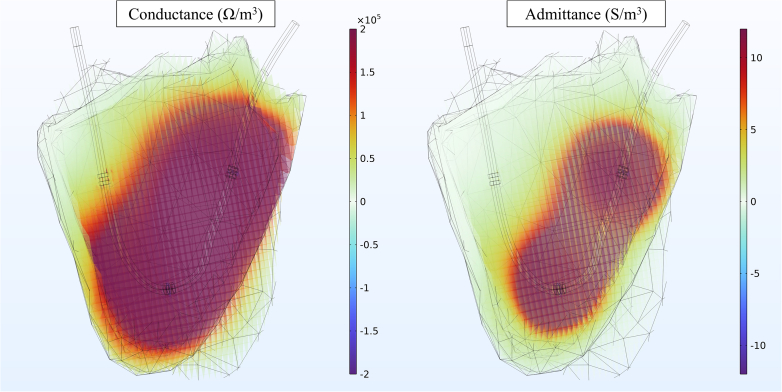
Finite Element Analysis Admittance vs Conductance Myocardial Sensitivity Plots ReZ sensitivity plot (A) and Gb sensitivity plot (B) through an admittance PAC within an RV model.

Finite element analysis (FEA) allowed us to determine the general area of increased admittance sensitivity inside the RV (Figure 2). This area does not span the entire chamber, but is concentrated in the RVOT. However, as shown previously by us,^16^ even if the main area of electric field sensitivity does not span the entire chamber, full chamber volumes can be accurately calculated by utilizing an independent measure of SV as a calibration factor for the admittance-to-volume equation (Equations 1 and 2). Chuong’s work^15^ further informed the additional partition of the input SV for the modified Wei’s equation to 60% of the thermodilution SV because the RVOT provides the largest percentage of maximal fractional area reduction during systole. Even though the admittance area of sensitivity is focused in the RVOT, we are able to calculate total RV volumes.

### FEA validation of admittance compared to conductance

FEA was utilized to compare the admittance technique performance against traditional conductance techniques for volume measurements. As described by Diaz Sanmartin et al,^16^ we can study the calculation of full-chamber volumes even if the admittance of regional sensitivity does not span the entire length of the ventricle. FEA models were created in COMSOL Multiphysics version 5.5, AC/DC module. A parametric model of the heart, including right and left atria and ventricles was developed to provide a simplified morphology. Material properties of both blood and muscle included both conductivity and permittivity values for each volume domain. Electrode pairs were modeled inside the RV, 1 pair at the RV apex and the second pair immediately below the pulmonary valve.

Given that an optimized tetrapolar configuration inside the RV only spanned the length of the outflow tract, we studied the electric field distribution in the RVOT to determine the admittance PAC spatial sensitivity. The computational models and sensitivity testing are based on the Geselowitz relationship as well as the divergence theorem regarding the volumetric relationship between vectorial current density, electric conductivity, permittivity, and measured voltage to complex admittance distribution over the volume as described by Larson et al^9^ utilizing the equation:

(3) $$Y=\int \int \int \frac{J_{\varphi }\cdot J_{\psi }}{\left(\sigma +j\omega \epsilon \right)V^{2}}\;dv$$

Where the admittance phasor *Y* is calculated by the alternating current densities J_φ_ and J_ψ_, the domain values for conductivity σ, permittivity ε, and the potential difference between voltage probes V.

### Animal studies

All protocols were approved by the office of the Institutional Animal Care and Use Committee at The University of Texas Health Science Center at San Antonio and conducted in accordance with the Institutional Animal Care and Use Committee guidelines and regulations. Animals (n = 10) (Yorkshire) were premedicated with Telazol 3 to 5 mg/kg, via intramuscular administration (Zoetis Services LLC). Yorkshire animal weights (120-150 pounds) were chosen to mimic adult human heart dimensions. General anesthesia was induced and maintained throughout the procedure using isoflurane (1%-2%) on room air with supplemental O_2_ to maintain adequate oxygen saturation during which animals were intubated and mechanically ventilated. Surface electrocardiography leads, a rectal thermistor and peripheral venous catheters were placed. Heating blankets were used as needed to maintain a core body temperature 100 to 102.5 °F.

Baseline transthoracic echocardiography was performed on all animals to confirm no significant valvular pathology and normal biventricular function (Philips iE33). Doppler studies were performed on a subset of pigs (n = 8) at baseline and following insertion of the admittance PAC, which demonstrated that the presence of the modified catheter did not induce tricuspid regurgitation. Venous and arterial access was obtained via standard Seldinger technique. Sheaths were placed in the right common femoral vein, right internal jugular, and right or left common femoral artery, the latter for continuous hemodynamic monitoring. Femoral venous access was used to introduce an 8-F, 28-mm Fogarty occlusion catheter used for inferior vena cava occlusion (IVCO) (Edwards Lifesciences). Internal jugular access was used to introduce the admittance technology (AT)-PAC. A 5-F micromanometer pressure sensor (Transonic) was used to measure RV pressure. Once all sheaths were in place, intravenous unfractionated heparin was administered to obtain goal activated clotting time >250 seconds.

Fluoroscopic guidance was used to position all catheters, with final position of AT-PAC being determined when at least 2 pairs of admittance electrodes were within the RV outflow tract. Baseline filling pressures via a PAC were recorded, in addition to invasive arterial pressure. Thermodilution CO (TD CO) was obtained by injection of room temperature saline (in triplicate) using the Vigilance II monitor (Vig II, Edwards Lifesciences) display. Then, baseline PV loops were obtained in triplicate during muscle paralysis (intravenous vecuronium 0.01 mg/kg), with expiratory breath hold (15-20 seconds), and during IVCO.

#### Dobutamine protocol

Intravenous dobutamine was started at a dose of 5 μg/kg/min and after a 15 to 20 minute period of drug infusion, repeat hemodynamics and TD CO were obtained using a PAC. Then, PV loops were obtained in triplicate with IVCO for each acquisition. Dobutamine was then turned off and after a 15- to 20-minute period of drug wash-out. Confirming return to baseline hemodynamics, we proceeded to the next pharmacologic intervention.

#### Phenylephrine protocol

Intravenous phenylephrine was started at a dose of 2 μg/kg/min and after a 15- to 20-minute period of drug infusion, repeat hemodynamics, and TD CO were obtained using a PAC. Then, PV loops were obtained in triplicate with IVCO for each acquisition. Phenylephrine was then turned off and after a 15- to 20-minute period of drug wash-out. Confirming return to baseline hemodynamics, we proceeded with the cardiac injury protocol.

#### Cardiac injury protocol

The right coronary artery (RCA) was engaged with a standard 6-F hockey stick guiding catheter (Merit Medical Systems). Then, 0.125 g Contour™ PVA-microspheres (Boston Scientific) mixed in a syringe with 10 mL saline and 10 mL contrast was prepared. Microspheres were injected into the RCA, 1 mL at a time accompanied by a 10-mL flush of saline after each microsphere injection. Repeated injections were performed every 3 minutes. Concomitantly, we administered 10 mg of intravenous metoprolol at escalating doses, at times up to 50 mg IV, while collecting serial hemodynamics using a PAC (including TD CO) as well as continuous arterial BP monitoring. PV loops were obtained in triplicate with IVCO for each acquisition. Once CO had reached 50% of baseline or the mean arterial pressure began dropping below 65 mm Hg, the protocol was complete. Due to inherent variability from animal to animal, the amount of microsphere injection or metoprolol dose varied between subjects.

#### Cardiac magnetic resonance

As a volumetric gold standard, MR images were acquired in all pigs 1 to 2 days before the admittance studies. Images were acquired on a Siemens TIM Trio 3T MR system (Siemens Healthineers, Erlangen, Germany) using a 6-channel body receiver coil with a transmit spiral coil with corresponding channel elements. 3- and 4-chamber longitudinal, and short-axis cine images consisting of 30 frames each between 2 end-diastolic phases were acquired of the entire heart. Short axis cines were segmented and measured across all frames to obtain baseline absolute volume measurements of all phases, and ESV and EDV.

#### Heart casting

Heart casting was performed as a secondary volume standard. RV foam casts were created by injecting the RV chamber with self-expanding foam (Loctite) on ex vivo hearts extracted from the animals, post mortem, at 5 mm Hg.

### Statistical analysis

**Figure 3:**
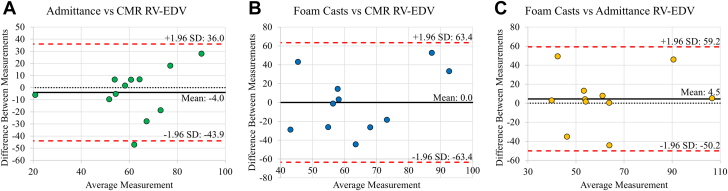
Bland-Altman Analysis Admittance volumes vs CMR (A), foam casts vs CMR (B), and foam casts vs admittance volume measurements (C). All measurements in mL. There is good agreement between admittance and the other 2 methods. RV = right ventricular; other abbreviations as in Figure 6.

Continuous data are presented as the mean ± SD unless otherwise specified. Two-tailed paired Student's *t-*tests were utilized to determine significant differences between hemodynamic parameters at baseline and during dobutamine, phenylephrine, and cardiac injury. Single factor analysis of variance was utilized to compare absolute volume parameters measured among the admittance PAC, CMR, and foam cast models. Figure 3 illustrates the agreement between volume measurement methods utilizing the Bland-Altman method.^17^

**Figure 4:**
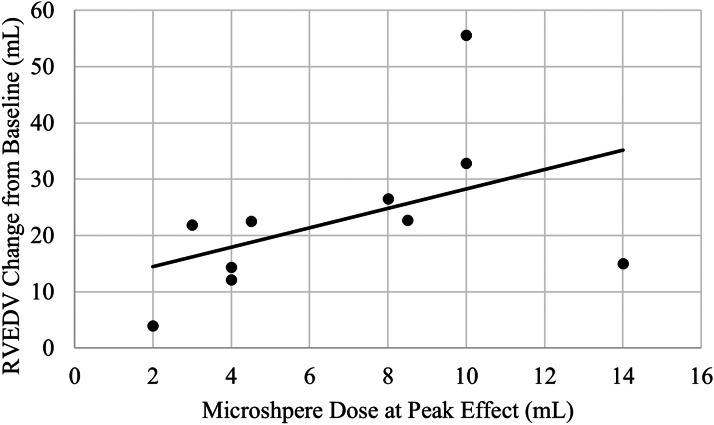
Acute RVEDV Dilation During Cardiac Injury (n = 10) The slope is not significantly different from zero (estimate = 1.7 ± 1.1; 95% CI: −0.9 to 4.3; *P =* 0.17); however, the change from baseline is significant (mean = 22.8 ± 4.5; 95% CI: 12.7-32.8; *P <* 0.001). RVEDV = right ventricular end-diastolic volume.

Data in Figure 4 were analyzed using the software SAS version 9.4 (SAS Institute). The hypothesis H_o_: μ = 0 vs H_1_: μ ≠ 0 was tested at α = 0.05, where μ is the mean value of the change in RVEDV from baseline to peak microspheres, with a 1-sample Student’s *t*-test. The association of the change in RVEDV from baseline to peak and escalating microsphere doses was analyzed using linear regression to determine the slope estimate with 95% CI.

## Results

### Catheter design

**Figure 5:**
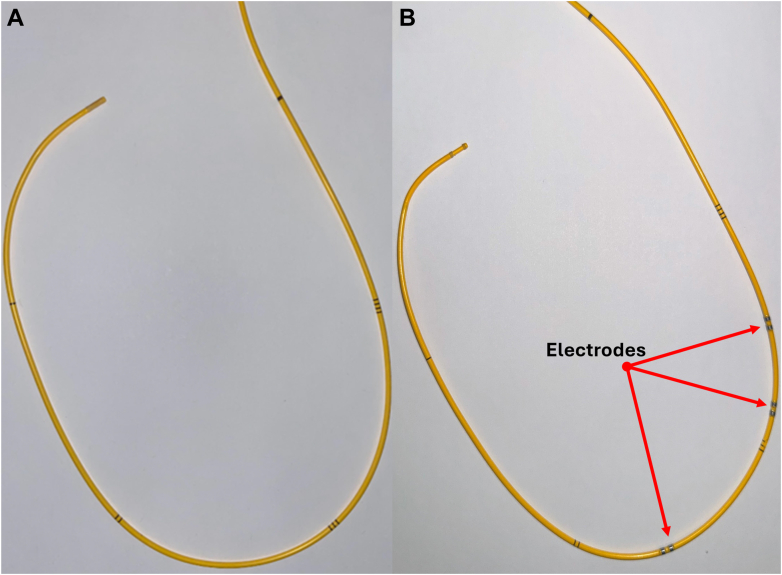
Normal and Modified Pulmonary Artery Catheter Designs Normal (A) and modified (B) pulmonary artery catheter designs.

Ex vivo studies of porcine heart right ventricular morphology were performed to obtain the optimal spacing between electrodes inside the chamber (n = 4). A final catheter design (Figure 5) was developed to account for the unique RV shape, maximizing the length between the electrodes placed in the RV apex and immediately below the pulmonic valve (7.5 cm). The admittance electrodes and wires were built into a 7-F PAC catheter, flanking the right atrial pressure port. For the purposes of the current study, the final catheter design included 2 pairs of electrodes at the RV apex and immediately below the pulmonic valve.

### Finite element analysis

FEA provided a S/m^3^ mapping of the RV parametric model admittance sensitivity. Admittance modeling (Figure 2) showed a significant improvement in preventing current leakage into the myocardium, localizing the measured signal within the RV blood chamber. When examining RVOT admittance measurements we found that the signal was confined to the outflow tract section of the chamber only and had minor admittance sensitivity to inflow RV volumes.

### Admittance volume validation

**Figure 6:**
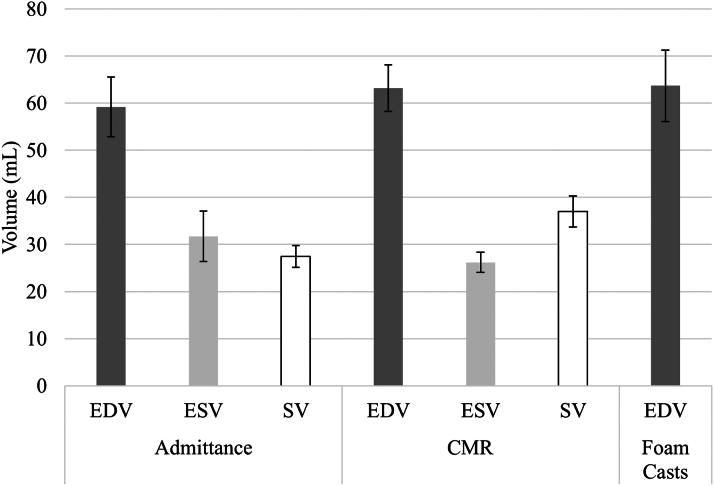
RV Volume Comparison Among Admittance, CMR, and Foam Casts *P =* NS. Data presented as mean ± SEM. CMR = cardiac magnetic resonance; EDV = end-diastolic volume; ESV = end-systolic volume; SV = stroke volume.

We found no significant variation in the mean EDV with this method (Figure 6) (EDV_Admittance_ = 59.2 ± 21.8 mL, EDV_CMR_ = 63.2 ± 17.2 mL, EDV_FoamCasts_ = 63.7 ± 26.3 mL, *P*_*ANOVA*_ = 0.87) and ESVs measured by admittance and CMR (ESV_Admittance_ = 31.7 ± 18.5 mL, ESV_CMR_ = 26.2 ± 7.33 mL; *P =* 0.32). Statistical difference was found between SV measured by the admittance PAC and CMR (SV_Admittance_ = 27.5 ± 8.05 mL, SV_CMR_ = 37.0 ± 11.44 mL; *P =* 0.002). Bland-Altman analysis showed low mean bias between admittance and the other 2 volume measurement methods, but wide 95% CIs (Figure 3). Comparing admittance to CMR volumes, the mean bias between the 2 methods was −4.0 mL (95% CI: −43.9 to 36.0 mL). Comparing admittance to RV foam cast volumes, the mean bias between the 2 methods was 4.5 mL (95% CI: −50.2 to 59.2 mL).

**Figure 7:**
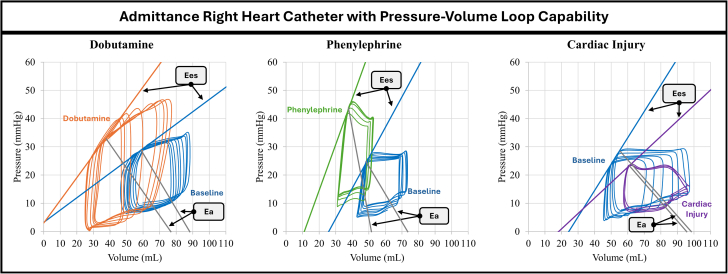
Right Ventricular Pressure-Volume Loop Comparisons Typical examples for each intravenous inotrope and intracoronary microsphere plus intravenous beta blockers are shown. The anticipated hemodynamic response is demonstrated. Ea = arterial elastance; Ees = end-systolic elastance.

Our results demonstrate the accuracy of the admittance technique’s anticipated PV-loop hemodynamic responses during IVCO (Figure 7, Table 1). Maximum RV volumes (RV Vmax) and EDVs decreased in response to dobutamine (RV Vmax_Baseline_ = 53 ± 22 mL, RV Vmax_Dobutamine_ = 39 ± 24 mL, *P*_*Vmax*_ = 0.011 and RVEDV_Baseline_ = 52 ± 21 mL, RVEDV_Dobutamine_ = 37 ± 22 mL, *P*_*EDV*_ = 0.014) and phenylephrine (RV Vmax_Baseline_ = 37 ± 26 mL, RV Vmax_Phenylephrine_ = 24 ± 17 mL; *P =* 0.029 and RVEDV_Baseline_ = 36 ± 26 mL, RVEDV_Phenylephrine_ = 23 ± 16 mL, *p*_*EDV*_ = 0.031 mL) (Figure 4). RV ESVs measured by the admittance PAC also decreased significantly in response to dobutamine (RVESV_Baseline_ = 28 ± 18 mL, RVESV_Dobutamine_ = 17 ± 10 mL; *P =* 0.020) (Table 1). The admittance PAC was also able to measure expected changes in calculated hemodynamic metrics, detecting a significant increase in Ea in response to phenylephrine (Ea_Baseline_ = 3.91 ± 3.73 mm Hg/mL, Ea_Phenylephrine_ = 8.26 ± 5.92 mm Hg/mL; *P =* 0.037), and a significant decrease of Ees under a cardiac injury model (Ees_Baseline_ = 1.7 ± 0.82 mm Hg/mL, Ees_Cardiac Injury_ = 1.12 ± 0.86 mm Hg/mL; *P =* 0.022).

**Table 1:** Hemodynamics Overview

	BL	Dob	*P* Value	BL	Phenyl	*P* Value	BL	MS	*P* Value
Heart rate, beats/min	95 ± 8	149 ± 11	0.001	93 ± 6	98 ± 11	0.13	89 ± 8	78 ± 11	0.051
ESP, mm Hg	27 ± 6	31 ± 12	0.56	25 ± 5	40 ± 4	0.002	28 ± 4	24 ± 4	0.018
EDP, mm Hg	13 ± 6	8 ± 4	0.15	14 ± 7	17 ± 6	0.080	14 ± 6	16 ± 6	0.31
Pmax, mm Hg	32 ± 5	42 ± 16	0.27	29 ± 5	46 ± 5	0.005	31 ± 5	26 ± 4	0.021
Pmin, mm Hg	8 ± 7	3 ± 4	0.057	10 ± 8	13 ± 6	0.007	9 ± 7	11 ± 7	<0.001
dP/dtmax, mm Hg/s	418 ± 136	1,272 ± 839	0.022	387 ± 175	695 ± 300	0.004	340 ± 147	211 ± 121	0.013
dP/dtmin, mm Hg/s	−292 ± 87	−556 ± 161	0.002	−275 ± 62	−457 ± 113	<0.001	−302 ± 89	−172 ± 59	0.04
Vmax	53 ± 22	39 ± 24	0.011	37 ± 26	24 ± 17	0.029	55 ± 43	60 ± 47	0.37
Vmin, mL	26 ± 18	13 ± 8	0.025	22 ± 17	13 ± 9	0.087	24 ± 19	29 ± 22	0.13
ESV, mL	28 ± 18	17 ± 10	0.020	23 ± 17	15 ± 13	0.12	26 ± 19	32 ± 24	0.13
EDV, mL	52 ± 21	37 ± 22	0.014	36 ± 26	23 ± 16	0.031	54 ± 43	57 ± 42	0.53
SV, mL	53 ± 5	53 ± 10	0.97	44 ± 15	60 ± 10	0.003	44 ± 8	36 ± 9	0.18
CO, L/min	5.1 ± 0.7	7.9 ± 1.4	0.001	4.1 ± 1.5	6.0 ± 1.5	<0.001	4.0 ± 1.0	2.8 ± 0.7	0.013
EF, %	47 ± 15	51 ± 12	0.63	35 ± 11	33 ± 12	0.43	46 ± 12	41 ± 7	0.035
SW, mJ	0.05 ± 0.02	0.09 ± 0.06	0.083	0.02 ± 0.01	0.03 ± 0.02	0.24	0.05 ± 0.04	0.03 ± 0.02	0.12
maxPwr, mW	12 ± 11	3 ± 2	0.076	9 ± 8	8 ± 6	0.66	11 ± 10	19 ± 15	0.087
Ea, mm Hg/mL	1.67 ± 1.79	2.06 ± 0.96	0.64	3.91 ± 3.73	8.26 ± 5.92	0.037	3.04 ± 4.3	2.16 ± 2.59	0.30
Ees, mm Hg/mL	1 ± 0.32	2.02 ± 1.29	0.076	1.93 ± 1.59	6.49 ± 5.09	0.093	1.7 ± 0.82	1.12 ± 0.86	0.022
PRSW, mm Hg	7.07 ± 8.29	21.02 ± 9.19	0.051	3.02 ± 4.27	5.85 ± 11.04	0.44	9.93 ± 4.1	5.92 ± 4.89	0.21
Ees/Ea ratio	0.98 ± 0.7	0.99 ± 0.39	0.88	0.8 ± 0.53	0.86 ± 0.39	0.86	1.46 ± 0.92	0.79 ± 0.58	0.13

Values are mean ± SD, unless otherwise indicated. Mean ± SD values are compared utilizing paired Student’s *t-*tests.

BL = baseline; CO = cardiac output; Dob = dobutamine; dP/dtmax = maximum first-derivative pressure; dP/dtmin = minimum first-derivative pressure; Ea = arterial elastance; EDP = end-diastolic pressure; EDV = end-diastolic volume; Ees = end-systolic elastance; Ees/Ea = coupling ratio; EF = ejection fraction; ESP = end-systolic pressure; ESV = end-systolic volume; maxPwr = maximum power; MS = microsphere-induced cardiac injury; Phenyl = phenylephrine; Pmax = maximum pressure; Pmin = minimum pressure; PRSW = preload recruitable stroke work; SV = stroke volume; SW = stroke work; Vmax = maximum volume; Vmin = minimum volume.

### RV dilation in response to cardiac injury protocol

During acute ischemia induced by intracoronary injections of microspheres, the admittance enabled PAC was able to identify a significant increase in measured RV EDV between baseline and escalating microsphere doses. As illustrated in Figure 4, the slope is not significantly different from zero (estimate = 1.7 ± 1.1; 95% CI: −0.9 to 4.3; *P =* 0.17); however, the change from baseline is significant (mean = 22.8 ± 4.5; 95% CI: 12.7-32.8; *P <* 0.001). The volume of microsphere injections necessary to induce RV EDV dilation varied between the n = 10 subjects, showing no relation between the number of doses injected and peak RV dilation observed (*P =* 0.12).

## Discussion

**Figure 8:**
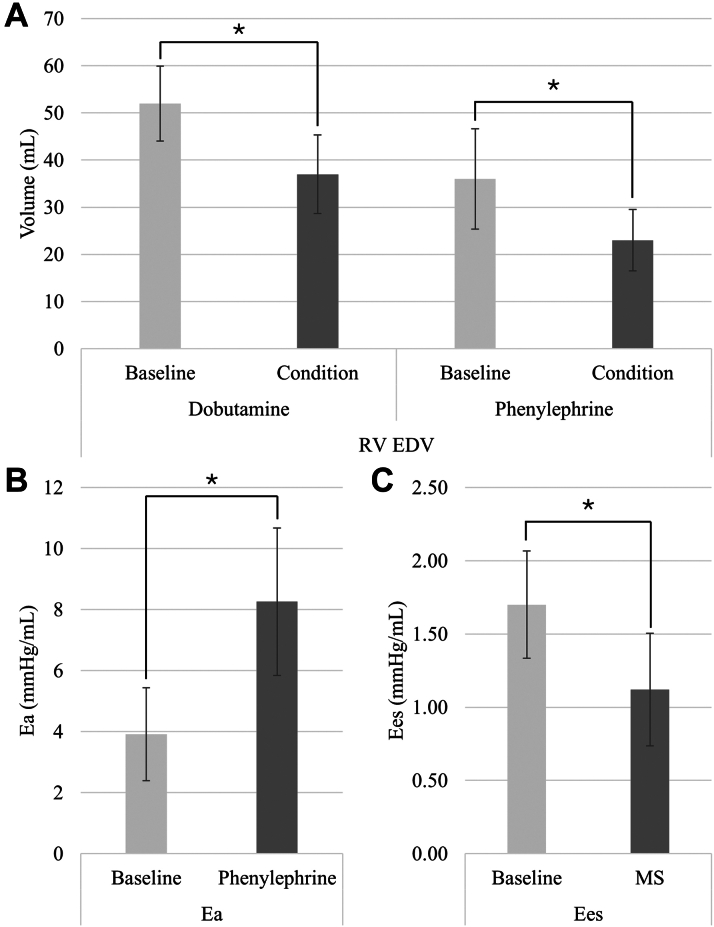
Hemodynamic Results of Admittance Pulmonary Artery Catheter (A) RVEDV comparison between baseline and dobutamine under positive inotropes (dobutamine and phenylephrine). (B) Ea comparison between baseline and phenylephrine. (C) Ees comparison between baseline and cardiac injury induced by intracoronary microspheres (MS). All changes are statistically significant (∗*P <* 0.05), data presented as mean ± SEM. Abbreviations as in Figures 4 and 7.

In our study, we were able to construct an admittance-enabled PAC with the ability to measure RV volumes accurately compared with other methods of chamber volume determination (ie, cardiac CMR and foam casting). Furthermore, the modified PAC was able to detect the expected physiological changes in response to dobutamine, phenylephrine, and cardiac injury (Figure 8). The real-time admittance volume signal also detected the increase in RV chamber volume that occurred with iterative myocardial injury in response to acute ischemia. For the first time, we demonstrate the ability to couple admittance technology with a PAC in a large animal preclinical model of relevant hemodynamic conditions and obtain clinically relevant data.

The clinical applications of such a modified PAC are myriad. Identifying subclinical RV dysfunction, or poor RV reserve in the context of unremarkable filling pressures, is an Achilles’ heel of invasive hemodynamics. Utilizing 2-dimensional echocardiography as a noninvasive and readily accessible modality has been common for managing patients’ pulmonary arterial hypertension (PAH) (World Health Organization Group I) patients. However, the current surrogate measurements for RV function in 2-dimensional echocardiograms, such as tricuspid annular plane systolic excursion, and others have clear limitations and have shown limited correlation with clinical risk stratification scores like the REVEAL 2.0 (Registry to Evaluate Early and Long-Term PAH Disease Management) risk calculator.^18^ Altering therapies for PAH patients hinges many times on static invasive hemodynamics. Because this patient population represents a captive audience who undergo serial right heart catheterization, assessment of RV chamber elastance, afterload, and RV-PA coupling at these time points routinely could be quite beneficial.^19^^,^^20^

Another subset of patients where understanding RV reserve is critical are patients undergoing LV assist device implantation. Despite many hemodynamic parameters and scores that are used to attempt to predict RV failure, it is still a significant clinical problem causing considerable morbidity and mortality.^21^^,^^22^

A relatively new frontier is tricuspid regurgitation.^23^^,^^24^ Although LV dimensions and the concept of proportionate vs disproportionate mitral regurgitation help guide these therapies for mitral regurgitation, such a framework is lacking for tricuspid regurgitation and the RV.^25^^,^^26^

**Figure 9:**
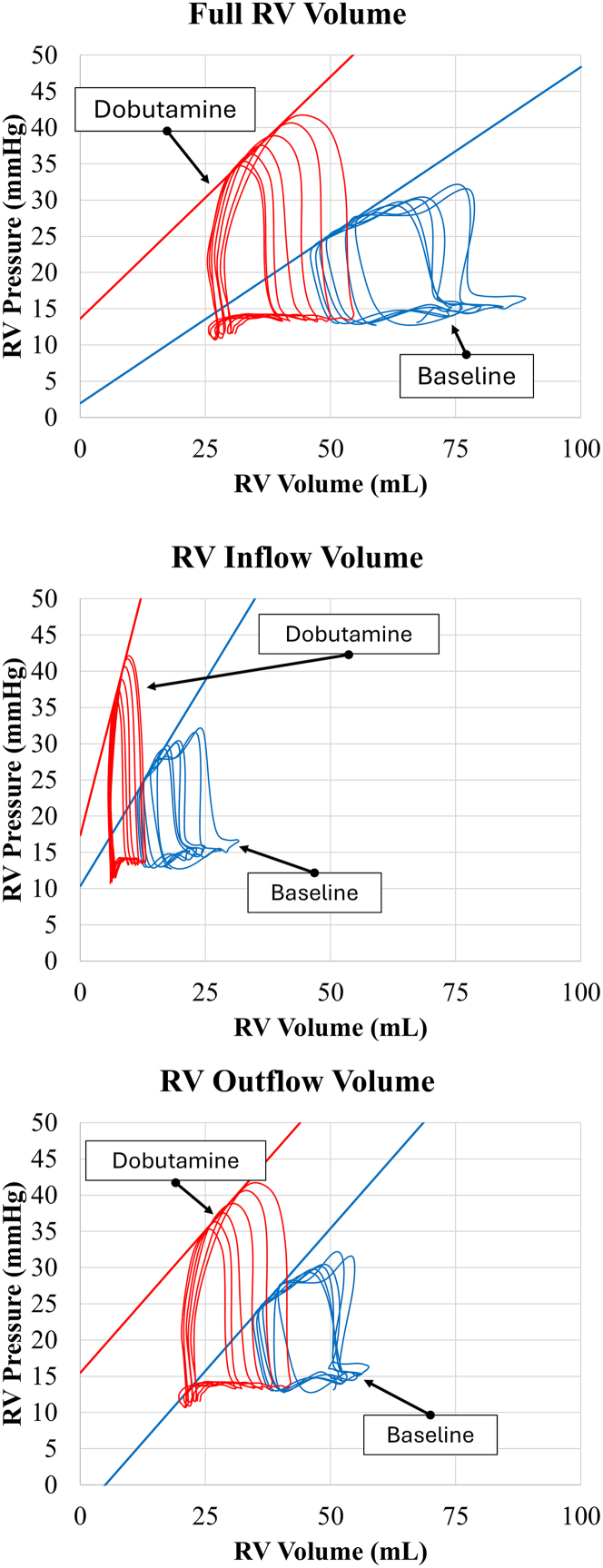
Inflow and Outflow RV PV-Loops Utilizing Modified Admittance Pulmonary Artery Catheter Data obtained from an early prototype admittance right heart catheter that can measure simultaneous right ventricular (RV) inflow, outflow, and combination (inflow + outflow) in a porcine study at baseline and during dobutamine infusion (5 μg/kg/min). RV pressure was obtained from a fluid-filled swan port.

One potential advantage of an admittance-enabled PAC is the inclusion of the RVOT volume. For example, utilizing biplane fluoroscopy, Chuong et al^15^ estimated that, in both a control state and during PA occlusions, the outflow tract showed a higher maximal fractional area reduction and maximal time rate of fractional area reduction during systole. They also showed that only RVOT deformation was affected by PA occlusion maneuvers (*P <* 0.05). In the current study, we only focused on the admittance signal of the RV outflow caused by limitations of the current admittance PAC design. However, we have begun to modify our current admittance PAC design, and demonstrate in Figure 9 the ability to obtain simultaneous RV inflow PV loops, RV outflow PV loops, and their combination to demonstrate what is possible.

It is known that acute ischemia results in ventricular dilation. Our model caused RV ischemia by injecting microspheres into the right coronary artery, leading to a linear dose-response regardless of sample size. We demonstrated the sensitivity of RV admittance by being able to detect this physiological change in real time (Figure 4). This feature of the modified PAC has an important advantage in monitoring and detecting chamber volume alterations on a minute-to-minute basis, potentially alerting the clinician to an impending clinical change.

### Study limitations

There are several limitations related to the current catheter design. First, as discussed in the Methods section, a hexapolar electrode configuration while performing a tetrapolar measurement limits the admittance sensitivity area to either the RVOT or the RV inflow tract. Through regional SV calibration this error was mitigated. Improved volume accuracy in future RV studies should focus on an RV volume catheter measuring the entire RV chamber. Regarding modifications to the catheter, the thermal dilution filament of the PAC used in this study was replaced by admittance wires to maintain a 7-F catheter profile. In future studies, the 7.5-F PAC will be utilized because it has an additional infusion port that could be utilized for admittance wires, maintaining the thermodilution capability of the PAC. The 7.5-F PAC will also allow use of the RV port to obtain simultaneous fluid-filled RV pressure. The addition of electrodes on the catheter plus accompanying wires through a lumen will increase catheter stiffness. Smaller diameter wires and electrodes can be utilized to address this concern. Regarding clinical translation, although the current study utilized IVC occlusions for multibeat analysis, studies in patients can use the Valsalva maneuver as well as external compressions of the inferior vena cava (pressure application on the skin), which have been used previously with success,^27^^,^^28^ or use of single-beat analysis for Ees.^6^ In a preliminary study (n = 1), we demonstrated that we were able to obtain comparable Ees values during external compression of the IVC in a pig at baseline (Ees_IVCO_ = 1.07 ± 0.1 mm Hg/mL, Ees_ExtCompression_ = 1.08 ± 0.09 mm Hg/mL; *P =* 0.90) and infusion of dobutamine (Ees_IVCO_ = 2.86 ± 0.06 mm Hg/mL, Ees_ExtCompression_ = 2.85 ± 0.06 mm Hg/mL; *P =* 0.82), each observation a mean of 3 consecutive family of PV loops.

Some limitations of cardiac MR and image analysis may have contributed to measurement disagreement during validation of CMR and the admittance PAC. Compared with 3D echocardiography, CMR has poorer temporal resolution, but better soft-tissue contrast. Studies have shown that although soft tissue contrast allows better delineation of anatomical structures, relatively low temporal resolution may obscure segmentation.^29^ This often presents as lower than expected EDVs and higher than expected ESVs, or a flattening of the volume curve. Still, CMR, being the gold standard for in vivo analysis of cardiac tissues and volumetry, provided the best modality on which to validate Admittance. Regarding Figure 3, although the mean bias between the admittance method and each reference (CMR, foam casts) was relatively small, the limits of agreement were wide, indicating substantial scatter. This suggests that although there may not be a strong systematic offset, the variability in measurements is large. Regarding the pairwise comparisons presented, results should be interpreted with caution, because type 1 error was not adjusted for multiple testing.

## Conclusions

We have demonstrated that it is feasible to obtain accurate instantaneous RV volume measurements utilizing an admittance electrode retrofitted PAC. Future studies will evolve the catheter to measure both inflow and outflow volumes and transition to use in patients from this preclinical model.

Perspectives **COMPETENCY IN MEDICAL KNOWLEDGE:** We demonstrated a new admittance PAC to measure RV PV loops for the first time. This includes accurate determination of absolute RV volumes, the expected responses to positive and negative inotropic maneuvers, and detecting acute RV dilatation in response to ischemia.

**TRANSLATIONAL OUTLOOK:** We have developed a new instrument with many clinical applications including determining which patients with tricuspid regurgitations have RV dysfunction and cannot tolerate the acute loss of the low-pressure leak; assist in management of patients with primary pulmonary hypertension during repeat right heart catheterizations; and predicting RV function post-LV assist device placement.

## Funding Support and Author Disclosures

This study was supported by grants from the National Institute of Health (R44/EB030973 and R44/HL145847 to Dr Feldman). Drs Valvano and Feldman have a financial relationship with CardioVol Corporation. Dr Feldman has received funding for research grants from Abiomed and CardioVol Corporation. All other authors have reported that they have no relationships relevant to the contents of this paper to disclose.
